# Trends and Determinants of Underweight among Children under Five Years in Ethiopia: Further Analysis with Ethiopian Demographic and Health Survey 2005–2016—Multivariate Decomposition Analysis

**DOI:** 10.1155/2022/6663756

**Published:** 2022-01-05

**Authors:** Tilahun Yemanu Birhan, Dessie Abebaw Angaw

**Affiliations:** Department of Epidemiology and Biostatistics, Institute of Public Health, College of Medicine and Health Science, University of Gondar, Gondar, Ethiopia

## Abstract

**Background:**

Underweight is one of the paramount major worldwide health problems, and it traces a big number of populations from infancy to old age. This study aimed to analyze the trends and predictors of change in underweight among children under five years in Ethiopia.

**Method:**

The data for this study were accessed from three Ethiopian Demographic and Health Survey data sets 2005, 2011, and 2016. The trend was examined separately for the periods 2005–2011, 2005–2016, and 2011–2016. Multivariate decomposition analysis of change in underweight was employed to answer the major research question of this study. The technique employed the output from the logistic regression model to parcel out the observed difference in underweight into components, and STATA 14 was utilized for data management and analysis.

**Result:**

Perceiving the overall trend, the rate of underweight was decreased from 38% in 2005 to 24% in 2016. The decomposition analysis results revealed that, about 12.60% of declines in underweight have been explained by the difference in population characteristics or endowments (*E*) over the study period. The size of the child at birth, husband's education, women's education, and household wealth index contributed significantly to the compositional decline in underweight.

**Conclusion:**

The magnitude of underweight among children under five years indicates a remarkable decline over the last ten years in Ethiopia. In this study, two-twelfth of the overall decrease in underweight among children under five years over the decade was due to the difference in characteristics between 2005 and 2016. Continuing to educate the population and boost the population's economy is needed on the government side in Ethiopia.

## 1. Introduction

Undernutrition is characterized as an obsessive condition that can be exacerbated by frequent infections, triggered by a lack of energy and protein in various areas [1]. It is one of the key global health issues, particularly in low- and middle-income countries [2, 3].

Undernutrition is one of the main signs of economic growth and contributes to a weakened immune system, the development of diarrheal disease, acute respiratory infection, and reduced cognitive and social development during childhood, as well as high blood pressure, diabetes, obesity, and heart disease in the adulthood [4–7].

In developing countries, the underweight burden is very high due to various factors such as large infant birth order, early childhood birth, low educational level, consanguineous marriage, low mother's body mass index (BMI), low iron intake during pregnancy, and low consumption of clean water [8–14].

Sub-Saharan Africa is home to the world's most nutritionally unpredictable population, owing to scarce conflict-related resources, HIV, tuberculosis (TB), and inadequate exposure to health services and environmental conditions, leading to the continent's overwhelming levels of underweight and food deficiency [9, 15–19].

Child health is considered an important predictor of socioeconomic growth; the key objective of Millennium Development Goal 4 (MDG) is to reduce mortality among children by two-thirds in 2015 [20]. However, no significant change in undernutrition has been perceived in Ethiopia, remaining one of the main causes of mortality and morbidity [3, 21, 22]. According to the most recent Demographic and Health Study, the impact of undernutrition may be a major concern for policymakers in Ethiopia, where around 5.8 million children under the age of five (38%) suffer from stunting and 24% suffer from being underweight [23]. While Ethiopia has been the fastest growing economy in the Horn of Africa, with 23% of the population living in underground poverty ($1.90 per day), it remains one of the poorest [23, 24]. While the prevalence of underweight and stunting in Ethiopia indicates some sort of decline over the past decades, it remains high for 22% of women of reproductive age to be undernourished, leaving their children predisposed to low birth weight, small stature, reduced infection resistance, and high risk of disease.

Studies are done previously utilized on point survey data [25–28], difficult to see the trends and identify factors consistently influencing underweight over time. In order to identify contributing factors associated with changes in underweight over time, the study of the shift in underweight using multivariate decomposition analysis is applicable to targeting interventions and to indicate clear programming to address underweight problems in Ethiopia. This study aimed to identify the determinant factors contributing to the change in the underweight rate over time using multivariate decomposition analysis based on the 2005, 2011, and 2016 Ethiopian Demographic and Health Survey (EDHS).

## 2. Methods and Materials

### 2.1. Study Design and Sampling

This study was based on a secondary analysis of cross-sectional population data from Ethiopia Demographic Health Surveys (EDHS) 2005, 2011, and 2016 to investigate trends and the factors associated with underweight among children under five years in Ethiopia.

In addition, in Ethiopia, four consecutive surveys were conducted in the cross-sectional years of 2000, 2005, 2011, and 2016. Similar to other demographic and health surveys, the principal objective of the Ethiopian Demographic and Health Survey (EDHS) was to offer current and consistent data on fertility and family planning behavior, child mortality, adult and maternal mortality, children's nutritional status, and use of maternal and child health services, as well as data, which were collected on knowledge and attitudes of women and men about sexually transmitted diseases and HIV/AIDS and evaluated potential exposure to the risk of HIV infection by exploring high-risk behaviors and condom use.

The sampling frame used for the 2016 EDHS was the Ethiopia Population and Housing Census (EPHC), which was conducted in 2007 by the Ethiopia Central Statistical Agency. The census frame is a complete list of 84,915 *enumeration areas* (EAs) created for the 2007 PHC. An EA is a geographical area covering on average 181 households. The sampling frame contains information about the EA location, type of residence (urban or rural), and an estimated number of residential households. Except for EAs in the six zones of the Somali region, each EA has accompanying cartographic materials. These materials delineate geographic locations, boundaries, main access, and landmarks in or outside the EA that help identify the EA. In Somali, a cartographic frame was used in three zones where sketch maps delineating the EA geographic boundaries were available for each EA; in the remaining six zones, satellite image maps were used to provide a map for each EA.

### 2.2. Variables and Measurement

The outcome variable for this study was underweight measured based on WHO guidelines, children under five years with weight-for-age *Z*-score of less than two.

Weight-for-age is a composite index of height-for-age and weight-for-height that accounts for both acute and chronic undernutrition. Children whose weight-for-age *Z*-score is below minus two standard deviations (−2 SD) from the median of the reference population are classified as underweight, while weight-for-age *Z*-score is above minus two standard deviations (−2 SD) considered as normal weight. Children whose weight-for-age *Z*-score is below minus three standard deviations (−3 SD) from the median are considered severely underweight.

The explanatory variables of interest in this study were as follows: child's age (months), child's sex, living area (urban/rural), mother's education level, and household socioeconomic status, place of delivery, antenatal care service during pregnancy, birth order, duration of breastfeeding, size of child at birth, BMI of women's, occupational status, vaccination status, and religion.

### 2.3. Statistical Analysis

This study employed a trend analysis of underweight among children under five years and decomposition of changes in underweight. The trend in underweight was analyzed using descriptive analyses, stratified by region, urban-rural residence, and selected sociodemographic characteristics. The trend was examined separately for the periods 2005–2011, 2005−2016, and 2011−2016. Data from EDHS 2005, 2011, and 2016 were appended together after extracting important variables for trend and decomposition analysis.

Multivariate decomposition analysis of change in underweight was employed to answer the major research question of this study. The purpose of the decomposition analysis was to identify the sources of changes in underweight in the last decade. Both changes in population composition and population behavior related to underweight are important. This method is used for several purposes in demography, economics, and other fields. The present analysis focused on how underweight responds to changes in children's characteristics at adult age and how these factors form differences across surveys conducted at different times. Both the difference in composition (Endowments) of the population and the difference in the effect of characteristics (coefficients) between the surveys are important to know the factors contributing to the decrease in underweight over the last ten years.

The multivariate decomposition analysis for nonlinear response utilizes the output from the logistic regression model (Binary outcome) to parcel out the observed difference in underweight into components. The difference can be attributed to compositional changes between surveys (i.e., the difference in characteristics) and changes in the effects of selected explanatory variables (i.e., the difference in the coefficients due to changes in population behavior).

Logit-based decomposition analysis technique was used to identify factors contributing to the change in underweight rate over the last decades. The observed difference in underweight between different surveys is additively decomposed into a characteristic (or endowment) component and a coefficient (or effect of characteristics) component. STATA 14 was utilized for data management and analysis, and STATA command with mvdcmp package was employed throughout the process of analysis. All calculations presented in this manuscript were weighted for sampling probabilities and nonresponse using the weighted factor included in the EDHS data. In the process of testing statistical significance or associations, with 95% confidence interval calculations), complex sampling procedures were considered. The detailed sampling procedure was presented in the full EDHS report [23, 29, 30]. For linear relations, the dependent variable is a function of a linear combination of predictors and regression coefficients, where *Y* = *F* (X *β*), where *Y* denotes the *N* × 1 dependent variable, *X* is an *N* × *K* matrix of independent variables, and *β* is a *K* × 1 vector of coefficients, where *A* and *B* represent EDHS 2016 and 2005, respectively.

The mean difference in *Y* between groups *A* and *B* can be decomposed as

(1) $$\begin{array}{c} Y_{A}-Y_{B}=F\left(X_{A}\beta_{A}\right)-F\left(X_{B}\beta_{B}\right). \end{array}$$

For our logistic regression, the logit or log-odds of underweight are taken as

(2) $$\begin{array}{c} \text{Logit}\left(A\right)-\text{Logit}\left(B\right)=F\left(X_{A}\beta_{A}\right)-F\left(X_{B}\beta_{B}\right)=\underset{E}{\underset{︸}{\left[F\left(X_{A}\beta_{A}\right)\;-F\left(X_{B}\beta_{A}\right)\right]}}+\underset{C}{\underset{︸}{\left[F\left(X_{B}\beta_{A}\right)-\;F\left(X_{B}\beta_{B}\right)\right]}}. \end{array}$$

The *E* component refers to the part of the differential owing to differences in endowments or characteristics. The *C* component refers to that part of the differential attributable to differences in the coefficients of effect [31].

## 3. Result

### 3.1. Characteristics of the Study Population

This section presents the distribution of individual characteristics of the study population for each survey in 2005, 2011, and 2016 EDHSs. In the three consecutive EDHS surveys, the higher percentage of women was found in the age group of 20−34 years.

Across the three EDHS surveys, the proportion of Orthodox Christians showed a slight decline, from 43% in 2005 to 35% in 2016, while the proportion of Muslims increased from 34% to 41% in the same year. Regarding the educational status of women, in the first two surveys, about three-quarters (79% in 2005 and 69% in 2011) were not educated, and 66% were not educated in 2016. The number of women pursuing primary education rose from 17% in 2005 to 28% in 2011 and 28% in 2016 (Table 1). In this study, we found that the level of use of institutional delivery services grew from 5% in 2005 to 27% in 2016 (Table 2).

### 3.2. Trend of Underweight

**Figure 1:**
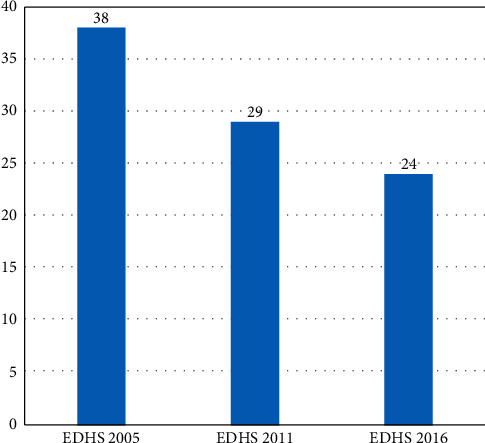
Trends of underweight among children under five years in Ethiopia, evidence from EDHS 2005–2016.

In this section, we present the underweight patterns over three consecutive periods of the EDHS survey. In order to see the difference in underweight over time and the main cause of the underweight shift, we split the trend duration into three stages, 2005–2011, 2011–2016, and 2005–2016. Perceiving at the overall trend, the rate of underweight has been declined over the last ten years (2005–2016), and the overall trend of underweight was decreased from 38% in 2005 to 29% in 2011 and 24% in 2016 (Figure 1).

Regarding certain background characteristics, the rate of decline is varied; it is clear that from 2011 to 2016, all regions experienced a decrease in underweight. Some groups have seen a substantial drop in underweight. With regard to the region, three regions, i.e., Tigray, Ethiopia's Southern Nations, Nationalities, and People's Region (SNNPR) and Amhara, from 2011 to 2016, recorded the greatest decrease in underweight among children under five. In terms of religion, a study showed that seven percentage points of the largest decrease in underweight over the last decade were seen among Orthodox Christians. In addition, during the second phase of the study period (2011 to 2016), rural residents reported the largest decrease in underweight, with a six percentage point decrease compared to urban ones with a one percentage point decrease in the same phase (Table 3).

## 4. Decomposition Analysis

### 4.1. Decomposition Analysis of Underweight in Ethiopia, 2005−2016

#### 4.1.1. Difference due to Characteristics Endowments (*E*)

Overall, there has been a significant decline in underweight rates in Ethiopia from 2005 to 2016. The decomposition analysis results revealed that about 12.60% of declines in underweight have been explained by the difference in women's characteristics or endowments (*E*) over the study period. Among the compositional factors, a significant contribution to the decline in underweight was associated with the size of the child at birth, husband's education, women's education, place of delivery, and wealth status while the duration of breastfeeding was contributed to the increment of underweight over time.

Thus, an increase in the composition of husband's attainment of primary and above education over the last ten years showed a significant contribution to the decrease in underweight (Table 4). In addition, the increase in the composition of women's attainment of primary education over the survey period showed a significant contribution to the decrease in underweight, it implies that education is needed to be the priority agenda to reduce the risk of underweight and other related morbidity and mortality among children. Similarly, an increase in the composition of household wealth quantiles over time (from 2005 to 2016) significantly contributed to the change in underweight (Table 3).

Besides, the increase in the composition of women's duration of breastfeeding (never breastfeed) over time (Table 1) has been significantly contributed to the increase in underweight (Table 3).

#### 4.1.2. Difference due to the Effect of Coefficients (*C*)

Controlling the role of change in compositional characteristics, 87.4% of the overall decline in underweight has been due to behavioral changes toward the improvement of child weight (Table 3). Factors significantly contributed to the change in underweight have been associated with household wealth quantile and age of women.

Keeping the effect of compositional factors constant, 21.3% of the decrease in underweight in the past decade was due to the change in behavior among household wealth quantile (Rich) (Table 3). Furthermore, keeping compositional factors constant, the behavioral change in the age of women between 20-34 and 35−49 has been significantly contributed to the decrease in underweight over the past decades (Table 3).

## 5. Discussion

One of Ethiopia's biggest public health challenges is undernutrition [19, 32]. Trends in child malnutrition in Ethiopia are determined by a large number of factors relevant to the treatment of infants and young children [32]. Despite this, Ethiopia has many dietary challenges; progress has been slow in reducing the prevalence of underweight in recent decades.

The aim of this study was to identify patterns and major compositional factors contributing to the change in underweight among children under the age of five over the last ten years in Ethiopia. We perceive a slight decrease in underweight among children under five years in Ethiopia in the past ten years; this decrease may be attributed to the government's great efforts to make the population aware of the importance of child care and feeding practices as well as improvements in female education [33,34]. Ethiopia is one of the sub-Saharan countries seeking to advance child and maternal well-being by challenging efforts to minimize high-level maternal and infant mortality through multicenter government and nongovernmental organization (NGO) nutrition programs linked to agriculture and nutrition [33,35,36]. In terms of residence, the percentage of underweight decreased at 4.06 percentage points among rural residents from 2005 to 2016. This may be attributed to the extension of global and national nutritional initiatives such as 2012 Ending Preventable Infant and Maternal Death [37], The Growth and Transformation Plan II (GTP II) (2015/16−2019/20), and the Seqota Declaration (2015) target to reduce hunger and undernutrition by 2030 and to encourage the launch of health infrastructure in rural areas which have been published in India in a similar report [24,38]. Therefore, they influence the continent's progress in food security by providing knowledge and advice on maternal nutrition during pregnancy, as well as education to resolve community myths and cultural attitudes about maternal and child nutrition through regular health contacts at health facilities [39].

The difference in coefficients (*C*) was responsible for two-twelfth of the overall shift in underweight, suggesting that a major contribution of the change occurs when population behavior changes through a significant explanatory variable consistent with a study conducted in Nepal and Malawi [40, 41]. Compositional change in wealth index makes an important contribution to the decrease in the underweight rate in Ethiopia over the last ten years, consistent with studies conducted in Vietnam and India [38, 40, 42] since community economy change will allow their children and mothers to access quality food during pregnancy as well as use a sustainable healthy environment for their children and the family in general.

In addition, compositional change in primary and higher education for husbands significantly contributed to the decrease in underweight over time among children under five in Ethiopia, in line with a study [43]. Educated people who are able to effectively follow all the food recommendations issued by health practitioners are more open to health infrastructure, are better connected to the media, and can have greater decision-making power to improve child health in the household and better financial capacity to care and feed children. Also, the compositional change in birth size has significantly contributed to the decrease in the underweight rate over time. This may be an infant born with a high birth weight with the potential to avoid infectious diseases and low infant morbidity, resulting in improved physical growth and psychomotor development; a similar outcome is published [14, 44]. In addition, an improvement in health facility delivery and female education over the duration of the study had a major impact on the decrease in underweight. This may be attributed to women receiving available therapy regarding the value of child feeding activities and treatment in health institutions. In addition, women's education had a positive effect on seeking health habits, managing families, providing their child a more healthy diet, and paying more attention to prenatal checkups; similar results are reported in Pakistan [14].

An infant who did not obtain breast milk from his mother had a substantial contribution to the rise in underweight. This may be attributed to children exposed to certain infectious diseases and unable to prevent it while a child consuming breast milk contributes to safeguarding adequate nutritional status, appropriate growth, and improved immunity for disease prevention in the child's body [45, 46]. In addition, breast milk significantly decreases the risk of morbidity and mortality from infectious diseases by eliminating the possibility of formula milk or other fluids and foods being infected [46, 47].

Controlling the effect of composition/population characteristics about 87.4% decrease in underweight over time was determined by the change in behavior of the population (effects). The influence of the wealth index and women's age was one of the striking results of the decomposition outcome of this study. Holding the roll of compositional variables steady as women get older leads substantially to the decline in underweight. This may be attributed to receiving experience in child care over time and being well educated of the ways in which their children are cared for and demonstrating good behaviors in terms of health and hygiene, breastfeeding, and inspired decision-making in child health.

The strength of this study was the analysis based on the nationally representative sample to ensure adequate generalizability of the study findings. The data employed were collected using a consistent standardized questioner, which provides an important source of information on underweight and nutritional status as well. The analysis technique used to facilitate the proportion of change in underweight over time into components attributable to changing socioeconomic and demographic characteristics of the population and change in underweight as well as the calculations was based on weight for the sampling probabilities and nonresponse. Further analytical techniques such as decomposition analysis were applied to recognize the source of change in underweight.

This study tries to highlight important findings to support nutritional programs in Ethiopia, but it is not without limitations, which may affect our conclusions. One of the limitations is its cross-sectional design, which limits the ability to make causal inferences evolve over time. The other limitation of this study was social desirability bias that creates problems for research and applied measurements.

## 6. Conclusion

The magnitude of underweight among children under five years slightly decreased over the last decades in Ethiopia. In this study, two-twelfth of the overall decrease in underweight among children under five years over the decade was due to the difference in characteristics between 2005 and 2016. Changes in composition of birth size, household wealth index, women's education, institutional delivery, and husbands' attainment of primary and higher education are attributable to the decline in underweight. Most importantly, the decline was due to the change in the behavior of handling underweight among the rich people and the age of women.

Continuing to educate the population is needed, as education is one of the major contributors to the decrease in underweight in Ethiopia. In addition, the government and anybody involved could concentrate on improving the economic status of households. Institutional delivery can be improved by accessing community health facilities and building awareness of nutrition.

## Figures and Tables

**Table 1:** Percentage distribution of sociodemographic characteristics among respondents from 2005–2016 EDHS.

Characteristics		2005 *N* = 8,123	2011 *N* = 9,496	2016 *N* = 8,755
Age of women	<20	25.10	6.21	22.28
	20–34	49.09	52.10	53.26
	35–49	25.82	23.66	24.46

Region	Tigray	6.15	6.72	6.63
	Afar	0.82	1.05	1.03
	Amhara	24.09	20.73	19.51
	Oromia	40.15	44.24	43.59
	Somali	3.56	2.65	4.30
	Benishangul-Gumuz	0.80	1.06	1.06
	SNNPR	22.37	21.02	20.83
	Gambela	0.26	0.32	0.24
	Harari	0.18	0.21	0.20
	Addis-abeba	1.26	1.69	2.24
	Dire-dawa	0.35	0.31	0.39

Religion	Orthodox	42.49	37.66	34.78
	Protestant	20.65	24.81	22.02
	Muslim	34.26	36.04	40.83
	Catholic	1.16	0.96	0.97
	Traditional	1.45	1.04	1.41

Husband's/partner's educational status	None	57.50	49.54	47.89
	Primary	31.35	42.67	40.20
	Secondary+	11.12	7.79	11.89

Women educational status	None	79.00	68.87	65.52
	Primary	16.78	27.68	27.57
	Secondary+	4.22	3.45	6.91

Wealth index	Poor	48.65	49.55	54.11
	Middle	16.86	16.36	14.46
	Rich	34.49	34.09	31.43

Residence	Rural	92.89	87.94	89.05
	Urban	7.11	12.06	10.95

Sex of child	Female	49.00	47.74	48.01
	Male	51.00	52.26	51.99

Size of the child at birth	Average and above	72.30	71.12	74.00
	Below average	27.70	28.88	26.00

Had diarrhea recently	Yes	18.57	13.73	11.67
	No	81.43	86.27	88.33

Birth order	1	19.65	19.48	19.81
	2–3	31.47	31.83	31.42
	4–5	23.07	23.00	23.34
	6+	25.81	25.69	25.43

Parity	1	12.93	12.45	13.28
	2–3	32.15	32.49	31.89
	4–5	24.91	25.09	24.98
	6+	30.01	29.97	29.85

Body mass index of respondents (BMI)	<18.51	19.70	21.83	19.83
	18.52–24.99	76.91	73.66	74.29
	25.00–29.99	3.39	4.51	5.87

Husband/partner occupational status	Working	99.33	98.03	89.28
	Not working	0.67	1.97	10.72

Women occupational status	Working	29.18	53.54	43.52
	Not working	70.82	46.46	56.48

**Table 2:** Percentage distribution of nutritional and health characteristics among respondents, 2005, 2011, and 2016 EDHS.

Characteristics		2005 *N* = 8,123	2011 *N* = 9,496	2016 *N* = 8,755
Stunting status	Stunted	47.41	43.77	38.48
	Not stunted	52.59	56.23	61.52

Ever had vaccination	Yes	61.28	76.14	65.63
	No	38.72	23.86	34.37

Place of delivery	Home	94.74	90.36	73.33
	H Institution	5.26	9.64	26.67

Duration of breastfeeding	Ever breastfed	50.11	52.38	51.30
	Never breastfed	4.27	3.64	5.25
	Still breastfed	45.61	43.95	43.45

Received vitamin A	Yes	45.93	52.38	35.76
	No	45.93	47.62	64.24

Number of antenatal care visits during pregnancy	0	70.53	57.28	35.98
	1–3	16.61	23.62	31.97
	4+	12.56	19.11	32.06

Last birth by cesarean section	Yes	0.96	1.18	1.83
	No	99.04	98.82	98.17

Anemic level of respondents	Anemic	29.47	18.54	30.16
	Nonanemic	70.53	81.46	69.83

**Table 3:** Trends in underweight among children under five years from 2005, 2011, and 2016 EDHS.

Characteristics	2005 *N* = 8,123	2011 *N* = 9,496	2016 *N* = 8,755	Percentage point difference in underweight		
				Phase I (2011–2005)	Phase II (2016–2011)	Phase III (2016–2005)
Age of women						
<20	30.00	27.50	29.10	−2.50	1.60	−0.90
20–34	28.18	30.50	24.40	2.32	−6.10	−3.78
35–49	31.00	31.63	26.63	0.63	−5.00	−5.63

Region						
Tigray	30.16	35.63	24.46	5.47	−11.17	−5.70
Afar	38.10	42.92	38.20	4.82	−4.72	0.10
Amhara	37.00	34.67	28.15	−2.33	−6.52	−8.85
Oromia	26.80	26.28	22.84	−0.52	−3.44	−3.96
Somali	30.00	31.38	26.88	1.38	−4.50	−3.12
Benishangul-Gumuz	36.28	33.28	33.39	−3.00	0.11	−2.89
SNNPR	26.00	29.08	20.75	3.08	−8.33	−5.25
Gambela	30.20	21.17	20.00	−9.03	−1.17	−10.20
Harari	33.33	20.45	19.69	−12.88	−0.76	−13.64
Addis-abeba	16.10	6.16	4.70	−9.94	−1.46	−11.40
Dirie-dawa	27.27	28.71	24.93	1.44	−3.78	−2.34

Religion						
Orthodox	28.00	28.67	22.00	0.67	−6.67	−6.00
Protestant	30.00	31.00	27.08	1.00	−3.92	−2.92
Muslim	25.36	24.00	21.27	−1.36	−3.27	−2.92
Catholic	30.88	33.64	28.20	2.76	−5.44	−2.68
Traditional	40.43	31.43	39.00	−9.00	7.57	−1.43

Husband/partner educational status						
None	35.08	35.28	31.14	0.20	−4.14	−3.94
Primary	25.80	27.21	21.53	1.41	−5.68	−4.27
Secondary+	17.00	14.96	15.44	−2.04	0.48	−1.56

Women educational status						
None	33.52	33.69	29.63	0.17	−4.06	−3.89
Primary	22.70	24.31	19.33	1.61	−4.98	−3.37
Secondary+	11.51	8.80	10.86	−2.71	2.06	−0.65

Household wealth quantile						
Poor	34.61	36.47	31.73	1.86	−4.74	−2.88
Middle	30.10	31.40	24.18	1.30	−7.22	−5.92
Rich	19.86	20.18	14.43	0.32	−5.75	−5.43

Residence						
Rural	31.60	33.00	27.54	1.40	−5.46	−4.06
Urban	15.44	14.77	13.55	−0.67	−1.22	−1.89

Sex of child						
Female	28.00	28.12	24.14	0.12	−3.98	−3.86
Male	30.00	31.87	26.00	1.87	−5.87	−4.00

Vaccination status						
Vaccinated	31.25	32.46	26.19	1.21	−6.27	−5.06
Not vaccinated	31.07	28.73	23.62	−2.34	−5.11	−7.45

Place of delivery						
Home	33.22	32.60	29.31	−0.62	−3.29	−3.91
H Institution	16.67	12.70	16.35	−3.97	3.65	−0.32

Duration of breast feeding						
Ever BF NK	31.00	32.25	27.61	1.25	-4.64	−3.39
Never breastfed	28.80	26.56	28.83	−2.24	2.27	0.03
Still breastfed	26.60	27.56	21.74	0.96	−5.82	−4.86

Size of a child at birth						
Average+	25.85	26.29	22.03	0.44	−4.26	−3.82
Below average	37.20	38.22	33.43	1.02	−4.79	−3.77

Birth order						
**1**	23.41	25.90	20.90	2.49	−5.00	−2.51
2–3	26.35	28.26	22.51	1.91	−5.75	−3.84
4–5	32.85	32.71	28.45	−0.14	−4.26	−4.40
6+	32.63	32.94	28.47	0.31	−4.47	−4.16

Parity						
1	20.53	25.32	18.01	4.79	−7.31	−2.52
2–3	26.02	27.31	22.50	1.29	−4.81	−3.52
4–5	32.24	32.38	27.87	0.14	−4.51	−4.37
6+	32.84	33.07	28.78	0.23	−4.29	−4.06

Anemic level in women						
Anemic	31.00	33.00	28.51	2.00	−4.49	−2.49
Nonanemic	28.00	29.13	23.26	1.13	−5.87	−4.74

History of ANC visit						
No	34.75	34.17	29.52	−0.58	−4.65	−5.23
Yes	21.73	27.55	20.13	5.82	−7.42	−1.60

BMI of women						
<18.51	37.80	39.22	34.31	1.42	−4.91	−3.49
18.52–24.99	27.70	28.00	23.73	0.30	−4.27	−3.97
25.00–29.99	25.00	19.66	23.01	−5.34	3.35	−1.99

Husband/partner occupational status						
Working	26.34	25.00	25.17	−1.34	0.17	−1.17
Not working	34.53	30.21	25.41	−4.32	−4.80	−9.12

Women occupational status						
Working	32.26	29.50	25.65	−2.76	−3.85	−6.61
Not working	33.56	30.51	24.26	−3.05	−6.25	−9.30

**Table 4:** Decomposition of change in underweight among children under five years in Ethiopia, 2005 to 2016.

Characteristics	Difference due to characteristics (E)		Difference due to coefficients (C)	
	Coefficient (95% CI)	Pct	Coefficient (95% CI)	Pct
Religion				
Traditional	1	1	1	1
Protestant	−0.000433(−0.00087134 6.2069*e* − 06)	0.2985	.00474(−.00046264 .0099345)	−3.2684
Muslim	0.001005(−0.00066041 0.0026708)	−0.6937	0.04848(−0.0060609 0.10301)	−33.455
Catholic	0.001920(−0.0022733 0.006114)	−1.3253	0.04847 (−0.0060609 0.10301)	−33.455
Orthodox	−0.00190(−0.0046687 0.00087657)	1.3085	0.09183 (−0.023772 0.20729)	−63.326

Husband education				
None	1	1	1	1
Primary	−0.00154(−0.0027481–0.00032363) ^*∗∗*^	1.06	−0.00894(−0.028397 0.010521)	6.1685
Secondary+	−0.00076(−0.0013856–0.00013266) ^*∗∗*^	0.5239	−0.00517 (−0.017381 0.0070503)	3.5647

Women education				
None	1	1	1	1
Primary	−0.00372(−0.0060008 −0.0014286)^*∗∗∗*^	2.564	−0.00482(−0.018073 0.0084313)	3.327
Secondary+	−0.00054(−0.001548 0.00047086)	0.3717	0.005503(−0.0025282 0.013534)	−3.7976

Wealth status				
Poor	1	1	1	1
Middle	−00023(−00040267 −0.000051783) ^*∗∗*^	0.1568	−0.00465(−0.019349 0.010048)	3.2095
Rich	−00061(−00088028 −0.00032973)^*∗∗∗*^	0.4175	−0.03091(−0.054916–0.0069075)^*∗∗*^	21.333

Residence				
Urban	1	1	1	1
Rural	0.000016(−0.0001251 0.0001569)	−0.0101	−0.01286(−0.13016 0.10443)	8.8757

Sex of child				
Female	1	1	1	1
Male	−0.000195(−0.00049505 0.00010555)	0.1344	−0.000435(−0.025461 0.024591)	0.3002

Place of delivery				
Home	1	1	1	1
H institution	−0.00442(−0.0087467–0.00008599) *∗*	3.048	0.00383(−0.0039667 0.011626)	−2.643

Duration of breast feeding				
Ever BF NK	1	1	1	1
Never breastfed	0.00021(0.00001349 0.00039271)^*∗∗*^	−0.1402	0.00578(−0.0027821 0.014344)	−3.9895
Still breastfed	−0.0019(−0.00017662 8.2208*e* − 06)	0.05811	−0.0226 (−0.062346 0.017125)	15.604

Size of child at birth				
Below average	**1**	1	1	1
Average+	−0.00041(−0.00059418 −0.00022529) ^*∗∗∗*^	0.2828	−0.00535(−0.021837 0.011143)	3.69

Parity				
1	1	1		
2–3	0.000092(−00071691 0.00089987)	-00.0631	0.00537 (−019997 0.030745)	−3.708
4–5	0.00060(−00024845 0.00056701)	-0.1099	0.00136 (−02083 0.023549)	−0.938
6+	−00003(−0008686 0.00081184)	0.01960	−00036 (−037947 0.037223)	0.2498

Anemic level				
Anemic	1	1		
Nonanemic	−00087(−00062717 0.00045323)	0.06002	−01624 (−057183 0.02471)	11.205

History of ANC visit				
No	1	1		
Yes	−00137(−0.0057163 0.002978)	0.9449	0.000268 (−018223 0.01876 −)	−0.185

Age of women				
<20	1	1		
20–34	−0.00221(−0.0049266 0.00051129)	1.5236	−10619 (−0.19881 −0.013565)^*∗∗*^	73.284
35–49	0.000284(−0.00010979 0.00067763)	−0.1960	−04901 (−094555 −0034679)^*∗∗*^	33.825

Constant			−0945(−0.41449 0.22544)	65.234
Total	−01824(−0.028988 −0074921)^*∗∗∗*^	12.60	−12666 (−15658 −096742)^*∗∗∗*^	87.40

^*∗*^Significant at 0.05, ^*∗∗*^significant at 0.01, ^*∗∗∗*^significant at <0.001, Pct = percentage contribution.

## Data Availability

The data sets used and/or analyzed during the current study are available in the Ethiopian Statistical Agency and Ministry of Health. The authors have submitted the proposed title and the aim of the paper to the online DHS website to download and use the data for this study. The EDHS program authorized accessing the data, and the data were used in the current study. The data are available at https://dhsprogram.com/Data/terms-of-use.cfm.

## Abbreviations

AIDS: Acquired immunodeficiency syndrome
ANC: Antenatal care
BMI: Body mass index
CSA: Central Statistical Agency
DBF: Duration of breastfeeding
EAS: Enumeration area
EDHS: Ethiopia demographic and health survey
EPHC: Ethiopian population and housing census
HIV: Human immunodeficiency virus
MOH: Ministry of Health
NGO: Nongovernmental organizations
SNNPR: Southern Nations, Nationalities, and People's Region
WHO: World Health Organization.
